# Green vs. Screen: Exploring the Outcomes of an In-Person and Virtual Nature-Based Environmental Education Intervention for Low-Income Children

**DOI:** 10.3390/su141912600

**Published:** 2022-10-04

**Authors:** Nadav L. Sprague, Ashby L. Sachs, Christine C. Ekenga

**Affiliations:** 1 Department of Epidemiology, Mailman School of Public Health, Columbia University, New York, NY 10032, USA; 2 Environmental Studies Department, University of Colorado Boulder, Boulder, CO 80309, USA; 3 Barcelona Institute for Global Health (ISGlobal), 08003 Barcelona, Spain; 4 Gangarosa Department of Environmental Health, Rollins School of Public Health, Emory University, Atlanta, GA 30322, USA

**Keywords:** COVID-19 pandemic, virtual learning, quality of life, nature exposure and immersive virtual nature, public health emergencies

## Abstract

The onset of the COVID-19 pandemic in 2020 forced a rapid transition to virtual learning. During the pandemic, many nature-based environmental education (NBEE) interventions shifted to virtual formats. In this study, we compare the impacts of a virtual NBEE intervention with its inperson NBEE counterpart. Between January and May 2021, a total of 49 low-income children (ages 9 to 13) from St. Louis, MO USA participated in this study. There were 37 children that participated in the virtual NBEE intervention and 12 students in the in-person NBEE intervention. Study participants completed a pre-/post-test survey that included items related to exposure to nature, perceived neighborhood safety, and self-reported quality of life. Children who participated in the in-person intervention reported higher post-intervention levels of nature exposure, perceived neighborhood safety, self-confidence, and self-efficacy than children who participated in the virtual intervention. The study’s findings have implications for the implementation of virtual learning during future public health emergencies and environmental disasters, including extreme climate events.

## 1. Introduction

In spring 2020, schools in approximately 191 countries worldwide quickly paused in-person instruction as a response to the COVID-19 pandemic. In an effort to mitigate the transmission of the COVID-19 virus through social distancing, many school districts shifted to online, virtual learning [1]. Many researchers and individuals drew parallels between traditional homeschooling and the emergency COVID-19 virtual learning; however, these two settings differ significantly as the emergency COVID-19 virtual learning was “school at home” led by teachers online rather than homeschooling led by primary caregivers [2]. Virtual learning is understood as the process of learning or receiving information with multimedia techniques, which may be independent of the time and space. During emergency COVID-19 virtual learning, students and teachers can interact through various means such as the internet or intranet [3–6]. There are several potential benefits to virtual learning, including flexible formats and opportunities for individualized learning curricula that may promote student motivation [7,8]. However, virtual learning has also been linked to significantly higher dropout rates than in-person learning, likely because of student isolation [9]. Student isolation has also been a common critique of traditional homeschooling [10].

In the United States (US), the transition to virtual learning during the COVID-19 pandemic highlighted and exacerbated many educational disparities [11–13]. For example, children from homes with fewer resources struggled to create successful virtual learning environments as they had less access to virtual learning necessities (e.g., computer, highspeed internet, and nutritious meals) and were exposed to more learning deterrents (e.g., harmful environmental exposures, death of a family member due to COVID-19, and distracting learning environments) than their higher-resourced counterparts [11,12,14]. Students with fewer opportunities for parental support, especially students in a single parent household, also risked falling further behind classmates [15,16]. These adverse learning outcomes suggest that the COVID-19 pandemic can be thought of as a ‘threat multiplier ’ for childhood educational disparities [13]. A threat multiplier for childhood educational disparities is a radical event of change that intensifies pre-existing educational disparities for children most at risk [17,18].

In addition to drastically changing children’s learning environment, studies suggest that the COVID-19 pandemic was a threat multiplier for disparities in access to nature-contact and time spent outside. A large body of evidence that was conducted prior to the COVID-19 pandemic documented racial and socioeconomic disparities in time that children spent outside [19–21]. Throughout the United States, many people increased their time outdoors during the COVID-19 pandemic in attempt to reduce the mental and physical health burden of the social distancing regulations [22,23]. Despite the increase of nature contact and time spent outside for the general population, studies suggest that some children actually experienced significant reductions in outdoor activities due to the COVID-19 pandemic [24]. One Canadian household survey (N = 1472) found that children saw significant declines in time walking, biking, playing sports, and outside during the COVID-19 pandemic [25]. Childhood reductions in nature contact and time outside were not uniform. Studies suggest that, in the US, Black children and children with lower markers of socioeconomic status saw greater reduction in time spent outside than their counterparts [13]. The increased nature contact inequity faced by Black children and children of lower socioeconomic status is worrisome as evidence suggests nature contact improves children’s wellbeing, perceived safety, and health.

A large body of studies and scientific reviews describe the positive effects of greenspace, nature contact, and time spent outdoors on child development, psychological wellbeing, and overall health [26–28]. Existing theories have focused on the inherent stress reducing influence of the natural world, how nature focuses attention, or how our evolutionary tendency prefer natural scenes may explain why nature may be beneficial for human health and wellbeing. The Kaplans’ Attention Restoration Theory has been a leading theory connecting nature to wellbeing [29]. According to Attention Restoration Theory, nature has the capacity to renew attention and promotes wellness through reduced mental fatigue. E.O. Wilson’s biophilia hypothesis looks to human evolution, arguing that humans possess an innate tendency to focus on life and lifelike processes [30]. Ulrich’s Stress Reduction Theory asserts that humans have automatic beneficial physiological responses in safe natural places [31].

Current research suggests that greenspace and perceived naturalness of an area is correlated with higher rates of perceived safety and violence prevention [32–34]. Spending time with others outdoors can reduce barriers between community members, enhance feelings of connectedness with others, and reduce stress levels [35,36]. Feeling safe in one’s community is a key component of mental wellness. Childhood fear of violence has been associated with negative effects on mental and physical health outcomes throughout the life course [37,38]. In addition, engaging with nature can promote increased quality of life in youth [39,40].

In considering connection to nature, many children are increasingly worried about planetary health. Research has found that growing eco-anxiety or climate change worry is significantly related to low well-being [41]. Youth stress was compounded during the prolonged COVID-19 lockdown, which amplified children’s feelings of anxiety and isolation. Children’s daily habits completely changed as they were forced to attend school virtually at home [42]. In addition, many outdoor educational programs were forced to pivot to provide nature-based online learning alternatives [43].

When considering nature-based online learning, questions may naturally arise as technology is often cited as the source of a growing disconnection between humans and the natural world [44]. However, in the context of events of threat multiplication such as the COVID-19 pandemic, US society has been reliant on technology [45]. Additionally, technology has created unique opportunities to interact with nature while indoors [46]. Both the COVID-19 pandemic and extreme weather events caused by climate change (such as the hot, smoke-filled wildfire seasons in the US) have brought virtual nature programming into focus as viable indoor alternatives to connect with nature. Peter Kahn and colleagues describe the concept of ‘technological nature’ as technologies that mediate, augment, and simulate our engagement with the natural world [47]. Researchers define ‘immersive virtual nature’ as the combination of visual and auditory stimuli that creates an immersive nature experience [48]. Emerging research suggests that a range of virtual nature experiences may have a positive influence on human health and wellbeing [49]. Virtual nature experiences could play a role in bringing the benefits of spending time in nature to cases where contact is not possible, for example, due to temperature excesses or individual disability [50].

This study investigates and compares the post-intervention exposures and health impacts of a virtual nature-based environmental education (NBEE) intervention with those of the well-established in-person NBEE intervention [40,51–53] in which it was modeled after. The study, conducted during the COVID-19 pandemic, included a sample of low-income children from St. Louis, Missouri USA. We evaluated the two NBEE interventions in terms of nature exposure, perceived neighborhood safety, and self-reported quality of life outcomes. We had four main research questions: (1) What are the outcomes of the two interventions in terms of children’s nature exposure? (2) What are the outcomes of the two interventions in terms of children’s perceived neighborhood safety? (3) What are the outcomes of the two interventions in terms of children’s quality of life outcomes? (4) Do the pre-intervention to post-intervention changes in children’s nature exposure, perceived neighborhood safety, and self-reported quality of life outcomes differ between the two types of intervention?

## 2. Materials and Methods

### 2.1. Study Context

A community-academic partnership between Columbia University’s Mailman School of Public Health, Emory University’s Rollins School of Public Health, Gateway to the Great Outdoors, and St. Louis Public School District (SLPS) was established in 2017 to provide a NBEE intervention for low-income children [13,39,40,52,53]. Since the community–academic partnership’s inception, it has provided free NBEE interventions to SLPS schools with 95% or more of its student body qualified for free or reduced lunch through the National School Lunch Program and the School Breakfast Program. Once the COVID-19 pandemic began in the United States, the community–academic partnership quickly pivoted to provide a virtual online adaptation to the NBEE intervention, commencing in January 2021. During this time, in St. Louis non-essential businesses were fully shutdown; however, people were allowed to go outdoors and to embrace nature. The community–academic partnership partnered with two SLPS classrooms that were teaching students through remote-online learning and one SLPS classroom that offered traditional in-person learning. Between January and May 2021, children that attended the in-person SLPS classroom participated in a traditional, in-person NBEE intervention (in-person intervention), and children that attended the remote-online learning SLPS classrooms participated in a virtual, on-line adaptation to the NBEE intervention (virtual intervention). This study was approved by the Institutional Review Board at Washington University in St. Louis, Emory University and Columbia University.

### 2.2. In-Person Intervention

The in-person NBEE intervention included both weekly interactive environmental education in-class lessons and monthly nature-based outings. Gateway to the Great Outdoors university student mentors lead weekly interactive environmental health-related S.T.E.A.M. (science, technology, engineering, art, and math) lessons in a small group setting. The in-person intervention’s weekly curriculum was developed by a team of community members with master ’s or doctoral degrees in biology, education, geology, public health, and environmental science. The weekly curriculum included of six units: Environment and Us, Ecosystems, Earth Systems, Climate Change, Healthy Lifestyles, and Community Based Projects. Each unit consists of five to six weekly lessons, in which the volunteer mentors selected from based on their students’ learning styles and interests. Monthly nature-based field trips augmented the weekly interactive lessons. Examples of monthly nature-based field trips include volunteering at urban farms, hiking in state parks, and canoeing down the Mississippi River. A more robust description of the 15 week in-person intervention is published elsewhere [39,40,52,53]. One SLPS classroom (12 students) participated in the in-person intervention.

### 2.3. Virtual Intervention

The community–academic partnership administered a virtual 15-week adaptation to the NBEE intervention to two SLPS classrooms (37 students). SLPS provided every student attending remote-online schools with Chromebooks. In lieu of in-person visits, Gateway to the Great Outdoors university student mentors would join SLPS virtual classrooms (either on Zoom or Google Classroom) and lead weekly lessons from the community-academic partnership’s weekly curriculum that were adapted to accommodate an online setting. To augment the weekly lessons, these classrooms participating in the virtual intervention attended monthly virtual-based nature field trips. Examples of the monthly virtual-based nature field trips include video chatting with park rangers to track box-turtles in Forest Park, taking virtual tours of national parks, and providing activities for children to do outside and then return to the session and share. All the virtual field trips either involved having students go out into nature or encouraged them embrace nature-contact after school hours.

### 2.4. Data Collection

We implemented a pre-test, post-test study design to evaluate changes in participant’s nature exposure, perceived neighborhood safety, and reported quality of life over 15 weeks. The survey also included questions on the child’s age, gender, race, ethnicity, and citizenship.

#### 2.4.1. Nature Exposure

In order to evaluate participant’s exposure to nature, we modified the validated Nature Exposure Scale II (NES II) [54] for use with middle school students. We chose the NES II to measure participant’s nature exposure as the validation study found this measure to have a very good reliability (α = 0.84) [54]. The following six subscales were included in our assessment: Daily time spent in nature; Attention to natural features; Amount of time traveling to nature; Attention to natural features while traveling; Time playing in nature; and Attention to natural features while playing. NES II subscale scores ranged from 1 (low nature exposure) to 5 (high nature exposure). We calculated an overall nature exposure score by summing all the subscales, ranging from 6 (low nature exposure) to 30 (high nature exposure). The Cronbach’s alpha was 0.83.

#### 2.4.2. Perceived Neighborhood Safety

To reduce the burden of the survey on the students, we used a one-question measure to evaluate perceived neighborhood safety. Perceived safety was accessed through the question “I feel safe walking in my neighborhood, day or night”. Students could answer either “very safe”, “safe”, “neutral”, “not safe”, or “very not safe”. Students who perceived their neighborhood as ‘very safe’ or ‘safe’ were combined into the category Safe, while those who perceived their neighborhood as ‘not safe’ or ‘very unsafe’ were combined as Unsafe. The Cronbach’s alpha was 0.79.

#### 2.4.3. Self-Reported Quality of Life

We utilized Pediatric Quality of Life Enjoyment and Satisfaction Questionnaire (PQ-LES-Q) to assess changes in self-reported quality of life [55]. We chose the PQ-LES-Q to measure self-reported quality of life as it is a validated measure with a high 1-week test–retest intraclass correlation coefficient of reliability of 0.78 [55]. The PQ-LES-Q consists of fifteen questions regarding the child’s quality of life during the past week. The following fifteen subscales were included in our assessment: Health; Mood and feelings; School and learning; Helping out at home; Getting along with friends; Getting along with your family; Play and free time; Getting things done; Your love and affection; Getting or buying things; The place you live; Paying attention; Your energy level; Feelings about yourself; and Your overall life satisfaction. PQ-LES-Q scores for each question range from 1 (very poor) to 5 (very good). The Cronbach’s alpha was 0.91.

### 2.5. Data Analysis

Our data analysis was twofold. First, we used chi-square and Wilcoxon signed-rank tests to evaluate within-group (pre-intervention versus post-intervention) differences in nature exposure, perceived safety, and quality of life scores for the in-person and virtual interventions separately. Then, we ran chi-square and Wilcoxon signed-rank tests to examine the between-group (in-person versus virtual) differences of the changes in nature exposure, perceived safety, and quality of life scores. All data analysis was conducted in R and RStudio.

## 3. Results

### 3.1. Intervention Participant Characteristics

A total of 49 SLPS students, 12 in the in-person classroom and 37 in the virtual classrooms, participated in the study. Table 1 presents the baseline demographic distributions of participants by intervention group. The in-person intervention’s mean age (12 years old) was significantly older than the virtual intervention’s mean age (10.0 years old; *p* = 0.001). As shown in Table 1, the racial composition of the two intervention groups were also significantly different, with the virtual group having a higher proportion of Black participants (*p* = 0.03). There were no other significant differences between these two groups.

### 3.2. Nature Exposure

Nature exposure scores are presented in Table 2. Prior to the intervention, there were no significant differences between the two groups in nature exposure scores (*p* > 0.05). Both groups experienced significant increases in daily time spent in nature, amount of time traveling to nature, and overall nature exposure (all *p* < 0.05). After the intervention, the in-person group had greater score increases than virtual group the for the following nature exposure subscales: Daily time spent in nature (in-person = +1.2, 95% Confidence Interval (CI): 0.8, 1.9; virtual = +0.5, 95% CI: 0.2, 0.6), Attention to natural features while traveling (in-person = +0.3, 95% CI: 0.1, 0.9; virtual = −1.0, 95% CI: −1.7, 0), and Time playing in nature (in-person = +1.4, 95% CI: 1.2, 2.1; virtual = +0.2, 95% CI: −0.1, 0.9) (all *p* < 0.05).

### 3.3. Perceived Neighborhood Safety

There were no significant differences in neighborhood safety prior to the intervention (*p* > 0.05). As shown in Table 3, the in-person intervention group experienced significant increases in perceived neighborhood safety (*p* = 0.048). The virtual intervention group also experienced significant increases in children that viewed their neighborhood as Safe (*p* = 0.001). There were also significant differences between groups in changes of perceived safety (*p* = 0.041).

### 3.4. Self-Reported Quality of Life

Table 3 displays the pre-intervention and post-intervention quality of life scores for both groups. There were no significant differences between the two groups in quality-of-life scores prior to the intervention (*p* > 0.05). The in-person group experienced significant score increases in the ‘Getting stuff done’ domain while the virtual group experienced significant score decreases in the ‘Feelings about yourself” domain (both *p* < 0.05). After the intervention, the in-person group had greater score increases than virtual group the for the following quality of life domains: ‘Getting stuff done’ (in-person = +1.3; virtual = −0.5), ‘Place that you live’ (in-person = +0.4; virtual = −0.3) and ‘Feelings about yourself’ (in-person = +0.4; virtual = −0.6).

## 4. Discussion

This study of low-income children during the COVID-19 pandemic examines the impacts of in-person and virtual nature-based environmental education interventions on nature exposure, perceived neighborhood safety, and self-reported quality of life. We observed that children who participated in the in-person intervention experienced more positive outcomes than children who participated in the virtual intervention. We discuss our findings in the context of current literature and implications for virtual learning during future public health emergencies and environmental disasters.

### 4.1. Nature Exposure

The NBEE interventions evaluated in this study countered some of the reported COVID-19-related trends in time spent outdoors and using screens. Both groups in our study experienced increases in the nature contact domains of daily time spent in nature, amount of time traveling to nature, and overall nature exposure. The in-person intervention was significantly better for promoting daily time spent in nature, time playing in nature, and attention to natural features while traveling than the virtual intervention. Prior reports have documented stark declines in childhood nature contact due to the COVID-19 pandemic [13,24]. As such, the two NBEE interventions may have acted both as a buffer to reduce the COVID-19 pandemic-related declines in childhood nature contact and as an effective intervention in promoting nature contact. Research conducted prior to the COVID-19 pandemic discusses NBEE interventions acting as a buffer for age-related declines in S.T.E.A.M. interest and seasonal declines in children’s health and wellbeing [52,53]. This observation is important as emerging research suggests that children who spent most of their time at home during the COVID-19 pandemic experienced better mental health when they were exposed to nature [56].

### 4.2. Perceived Neighborhood Safety and Self-Reported Quality of Life

There were significant increases in perceived neighborhood safety for both intervention groups; however, participants of the in-person intervention experienced greater improvements in perceived neighborhood safety than their virtual intervention counterparts. Considering that natural areas may be associated with higher rates of perceived safety [33], we hypothesize that the differences in post-intervention neighborhood safety perceptions may be due to direct exposure to natural settings, with the virtual intervention providing an inherently inferior virtual experience. This is supported by evidence which suggests that virtual nature is unable to match nature contact’s full range of mental, physical, emotional, psychosocial, and health benefits [46].

During the study period, we observed significant differences between the two intervention groups in the ‘Getting stuff done’, ‘Place that you live’, and ‘Feelings about yourself’ quality of life domains. The in-person intervention group experienced significant score increases while the scores for the virtual intervention group decreased. The COVID-19 pandemic served as an extraordinarily stressful experience for children and youth worldwide. Our results support the findings of previous studies that virtual learning environments were less effective at improving participants feelings about themselves. The lack of effectiveness is perhaps due to increases in loneliness and subsequent stress incurred by students during pandemic lockdowns [57]. The pandemic may also have exacerbated anxiety symptoms among children [58]. It is unsurprising that youth felt less productive in the virtual learning environment. This reflects sentiments reported in a large cross-sectional survey with American youth who described a lack of motivation, loss of focus, and reduced productivity while learning or working at home [59]. Distractions in the home and a lack of structure could have contributed to challenges in ‘Getting stuff done’ and negative feelings during the pandemic for virtual learners. The results of this study indicate that more work is needed to understand and improve the virtual learning experience for children.

### 4.3. Strength and Limitations

There were several strengths to this study, including longitudinal data collection at two time points during the COVID-19 pandemic and the use of validated measures of nature exposure and quality of life. Limitations of the study include the use of a convenience sample with a small sample size, and a narrow study period of 15 weeks. COVID-19 mitigation strategies differed between and within countries. Thus, findings may not be generalizable, particularly to non-US children. Our sample of low-income children was diverse. However, the intervention groups were not racially comparable as Black and Asian children comprised more than three-quarters of the virtual participants compared with the 33% Black and Asian composition of the in-person intervention group. Pandemic-related restrictions on outside visitors limited our ability to recruit more classrooms with non-White children for the in-person intervention. As a result, our sample was too small to investigate the influence of race and ethnicity on study outcomes. Nevertheless, these exploratory findings are worthy of further study, as study results should generate further interest in the impacts of virtual learning, specifically impacts by the type, frequency, and duration of virtual engagement. Next steps should include conducting qualitative focus groups of participants and classroom teachers to understand what potential mechanisms make the in-person intervention more effective than the virtual one in promoting perceived neighborhood safety, self-confidence, and self-efficacy. The qualitative investigation could further inform our results and allow us to provide recommendations to practitioners on how to enhance virtual NBEE interventions.

### 4.4. Conclusions

In this study, we observed that the virtual nature learning environment was less effective in promoting perceived neighborhood safety, self-confidence, and self-efficacy than the in-person learning environment. While lockdowns and other social distancing measures associated with the COVID-19 pandemic may be lifted, we suspect that future public health emergencies and environmental disasters will present similar challenges. Our study outcomes focused on perceived nature and safety exposures. However, other environmental exposures are worthy of investigation, in addition to health outcomes beyond health-related quality of life. Climate change, in particular, should be of concern as a potential threat multiplier [15]. Climate change is projected to make individuals spend less time in nature and more time indoors due to increased temperatures, decreased air quality, and increased extreme weather events [60]. Children have already spent increasingly less time outdoors than previous generations since the 1990s, and the decline is expected to grow exponentially due to climate change [61,62]. As a result, nature-based environmental education programs could become more reliant on virtual platforms to deliver curriculum [63]. Thus, in addition to immediate climate change mitigation, more work will be necessary to understand and overcome the limitations associated with virtual learning environments. Next steps include qualitative studies to better understand the pedological mechanisms for the differences in outcomes of the in-persona and virtual intervention.

## Figures and Tables

**Table 1.** Baseline demographic characteristics of participants by intervention.

	Total (N = 49)	In-School Intervention (N = 12)	Virtual Intervention (N = 37)	*p*-Value *
Age (Mean (Sd))	10.4 (1.18)	12.1 (0.6)	10.0 (0.7)	**0.001**
Female (N (%))	18 (36.7)	4 (33.3%)	14 (37.8%)	0.984
Hispanic (N (%))	7 (14.3)	2 (16.7%)	5 (13.5%)	0.954
Race (N (%))				**0.030**
Black	26 (53.1)	4 (33.3%)	21 (56.7%)	
American Indian or Alaska Native	1 (2.0)	0 (0.0%)	1 (2.7%)	
Asian	7 (14.3)	0 (0.0%)	7 (18.9%)	
Native Hawaiian/Pacific Islander	2 (4.1)	0 (0.0%)	2 (5.4%)	
White	4 (8.2)	3 (25.0%)	1 (2.7%)	
Two or More Races	5 (10.2)	3 (25.0%)	2 (5.4%)	
Self-Identified as Other	4 (8.2)	2 (16.7%)	2 (5.4%)	
Us Citizenship Status (N (%))				
Participant	42 (85.7)	11 (91.7%)	31 (83.7%)	0.839
Mother Of Participant	36 (73.4)	9 (75.0%)	27 (73.0%)	0.977
Father Of Participant	32 (65.3)	9 (75.0%)	23 (62.2%)	0.643

* *p*-value were calculated with chi-squared test for categorical variables and Wilcoxon signed rank for continuous variables for differences between groups.

Bold numeric values signify statistical significance.

**Table 2.** Nature Exposure Pre-Intervention and Post-Intervention Scores, by intervention group (n = 49).

	In-School Programming (N = 12)			Virtual Programming (N = 37)			Difference in Change between Groups

	Pre-Intervention	Post-Intervention	*p*-Value *	Pre-Intervention	Post-Intervention	*p*-Value *	*p*-Value **
Daily Time Spent in Nature	1.3 (1.0)	2.5 (1.2)	**0.006**	1.9 (1.4)	2.4 (1.4)	**0.0029**	**0.049**
Attention To Natural Features While in Nature	2 (1.3)	2.1 (0.9)	0.83	2 (1.2)	2.4 (1.3)	0.225	0.603
Amount Of Time Traveling to Nature	2.2 (1.0)	3.3 (1.0)	**0.011**	2.3 (1.3)	2.8 (1.2)	**0.002**	0.098
Attention To Natural Features While Traveling	2.8 (1.3)	3.1 (1.2)	0.491	2.5 (1.3)	1.5 (0.9)	0.309	**0.049**
Time Playing in Nature	2.3 (1.1)	3.7 (1.1)	**0.049**	2.8 (1.4)	3.0 (1.4)	0.094	**0.048**
Attention To Natural Features While Playing	1.8 (1.3)	1.9 (1.2)	0.871	1.8 (1.4)	1.9 (1.5)	0.792	0.691
Overall Nature Exposure	12.3 (4.0)	15.3 (3.4)	**0.048**	12.9 (4.2)	14.4 (4.4)	**0.042**	0.366

* *p*-value were calculated with Wilcoxon signed rank for continuous variables for differences within groups.

** *p*-value were calculated with Wilcoxon signed rank for continuous variables for differences between groups.

Bold numeric values signify statistical significance.

**Table 3.** Neighborhood safety and Quality of Life Pre-Intervention and Post-Intervention Scores, by intervention group (n = 49).

	In-School Programming (N = 12)			Virtual Programming (N = 37)			Difference in Change between Groups

	Pre-Intervention	Post-Intervention	*p*-Value *	Pre-Intervention	Post-Intervention	*p*-Value *	*p*-Value **
Perceived Neighborhood Safety			**0.048**			**0.001**	**0.041**
Safe	3 (25.0%)	6 (50.0%)		14 (37.8%)	16 (43.2%)		
Neutral	4 (33.3%)	5 (41.7%)		4 (10.8%)	8 (21.6%)		
Not safe	5 (41.7%)	1 (8.3%)		19 (51.4%)	13 (35.1%)		
Self-reported quality of life							
Health	4.7 (0.7)	4.8 (0.8)	0.28	4.1 (1.0)	4.0 (1.0)	0.490	0.658
Mood	3.7 (1.1)	3.8 (1.1)	0.83	3.6 (1.3)	3.7 (1.2)	0.452	0.916
School and learning	4.3 (0.6)	4.5 (0.6)	0.067	3.6 (1.2)	3.8 (1.2)	0.445	0.916
Helping out at home	3.8 (0.6)	4.4 (0.6)	0.056	3.7 (1.4)	3.9 (1.3)	0.3404	0.37
Getting along with friends	4.2 (1.1)	4.4 (1.2)	0.588	3.4 (1.7)	3.8 (1.1)	0.257	0.587
Getting along with family	4.1 (0.9)	4.3 (0.6)	0.503	3.8 (1.3)	3.9 (1.3)	0.643	0.242
Play and free time	4.3 (0.9)	3.8 (0.8)	0.096	3.7 (1.4)	3.5 (1.1)	1	0.615
Getting things done	3.5 (0.5)	4.8 (0.9)	**0.044**	4.1 (1.0)	3.6 (1.1)	0.357	**0.039**
Love and affection	3.3 (1.3)	3.6 (1.5)	0.24	4 (1.2)	3.9 (1.0)	0.329	0.441
Getting or buying things	2.9 (1.6)	3.5 (1.4)	0.223	3.4 (1.2)	3.6 (1.1)	0.117	0.559
Place that you live	4.4 (0.7)	4.8 (0.6)	0.14	3.8 (1.4)	3.5 (1.0)	0.822	**0.037**
Paying attention	3.8 (0.8)	3.7 (0.8)	0.674	3.6 (1.2)	3.6 (1.1)	1	0.881
Energy level	4.2 (1.0)	3.6 (1.3)	0.191	3.7 (1.5)	3.7 (1.1)	1	0.202
Feelings about yourself	3.6 (1.4)	4.0 (1.1)	0.339	3.4 (1.4)	2.8 (1.1)	**0.040**	**0.041**
Overall, how has your life been	4.3 (0.7)	4.5 (0.9)	0.503	4 (1.5)	4.1 (1.2)	0.920	0.809

* *p*-value were calculated with chi-squared test for categorical variables and Wilcoxon signed rank for continuous variables for differences within groups.

** *p*-value were calculated with chi-squared test for categorical variables and Wilcoxon signed rank for continuous variables for differences between groups.

Bold numeric values signify statistical significance.

## Data Availability

The data generated during the study are available from the corresponding author upon reasonable request.
